# Supplementary material for: Experimental demonstration of highly reliable dynamic memristor for artificial neuron and neuromorphic computing
Source: Nat Commun. 2022 Jun 3;13:2888. doi: 10.1038/s41467-022-30539-6 (PMC9166790; doi:10.1038/s41467-022-30539-6)
Supplement: Supplementary file 1 — Supplementary Information [file 41467_2022_30539_MOESM1_ESM.pdf]

Supplementary Information

Experimental demonstration of highly reliable dynamic memristor for reliable artificial neuron and neuromorphic computing

*See-On Park[1†], Hakcheon Jeong[1†], Jongyong Park[1†], Jongmin Bae[1], and Shinhyun Choi[1]**

[1]The School of Electrical Engineering, Korea Advanced Institute of Science and Technology (KAIST), Daejeon 34141, Republic of Korea

[†] These authors contributed equally to this work.
* **Address correspondence to** Shinhyun Choi, The School of Electrical Engineering, Korea Advanced Institute of Science and Technology (KAIST), Daejeon 34141, Republic of Korea, Email: shinhyun@kaist.ac.kr

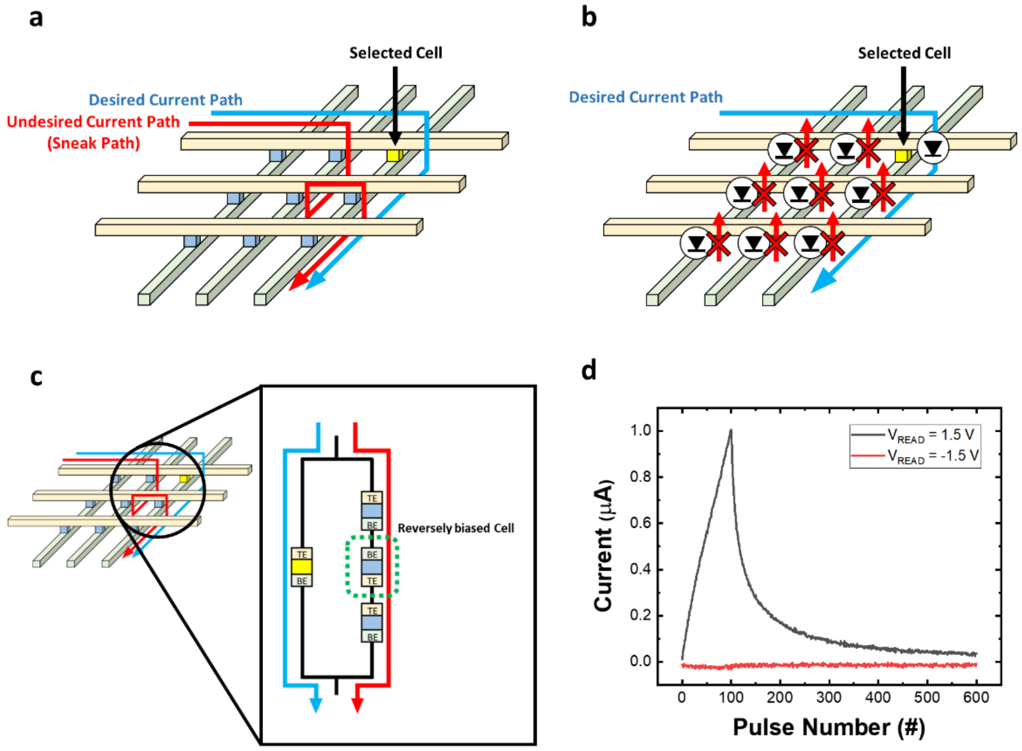

Supplementary Figure 1. Effect of self-rectifying characteristic in a memristor cross-bar array. (a) Schematic of a memristor cross-bar array without self-rectifying characteristic. The large leakage current is formed through numerous undesired current paths, called sneak paths. The sneak path interrupts the write/erase operation of memristor by interfering neighbor cells, and also interrupts read operation by making unselected cells to be read. **(b)** Schematic of memristor cross-bar array when memristors in a cross-bar array have the self-rectifying characteristic. The undesired current paths are blocked, thus the sneak path issues are resolved. **(c)** The diode-like characteristic of a memristor is effective because the sneak path must pass the reversely biased cell. **(d)** The pulsed response of the gradual TiO_x memristor in a 20×20 cross-bar array when is read by V_{read} (+1.5 V) and negative V_{read} (-1.5 V), respectively. The dramatic conductance change from consecutive 100 SET pulses and following dynamic self-decaying is clearly shown when the memristor is read by positive read bias. However, an extremely small current is sensed when the negative read voltage is used, which means that the leakage path cannot be formed in the gradual TiO_x memristor cross-bar array.

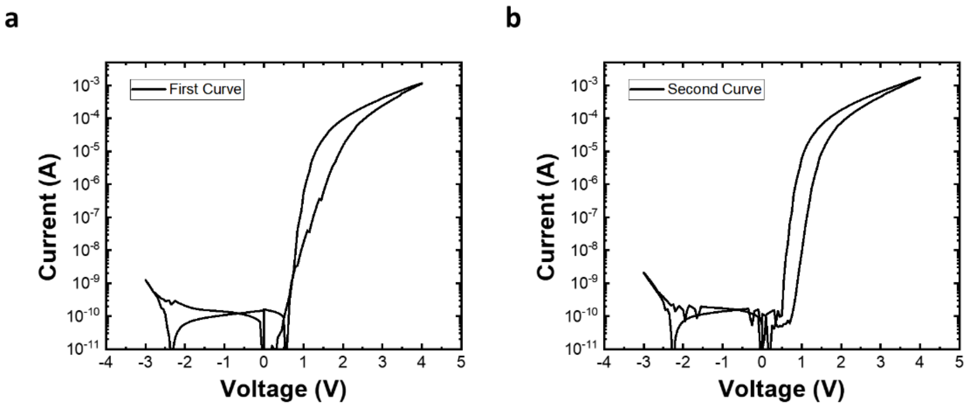

Supplementary Figure 2. Forming-free property of the device. (a) The first I-V curve of the gradual TiO_x memristor. **(b)** The second I-V curve of the device. The device did not show the necessity of the forming process, but the first I-V curve has little difference compared to the second curve. The shape of the first I-V curve in **(a)** only appears in the first voltage sweep, and only the shape of the second I-V curve appears after the first sweep.

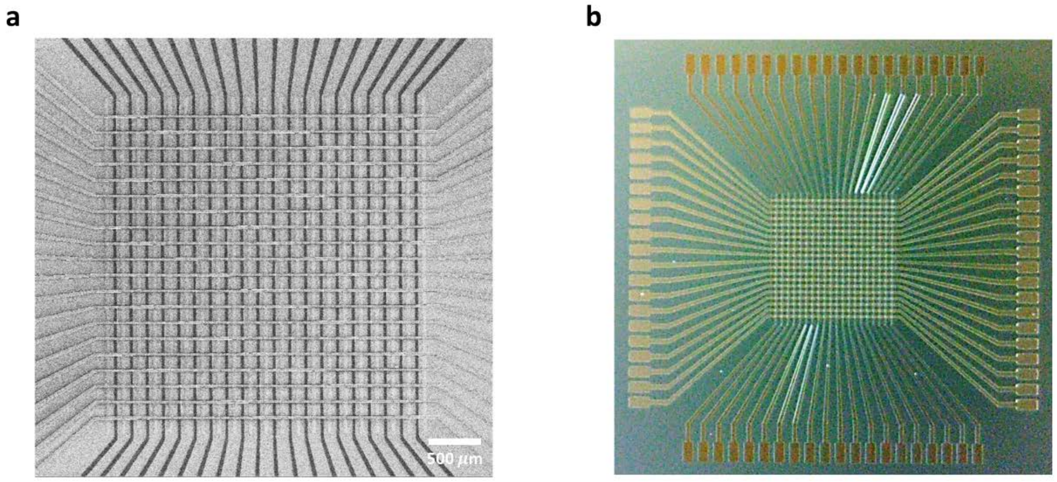

Supplementary Figure 3. A scanning electron microscope (SEM) image (a) and an optical microscope image (b) of the fabricated cross-bar array. The size of a single cell in the array is 5×5 μm2. The column lines are bottom electrodes (Ti) and the row lines are top electrodes (Pt). Scale bar in the SEM image: 500 μm

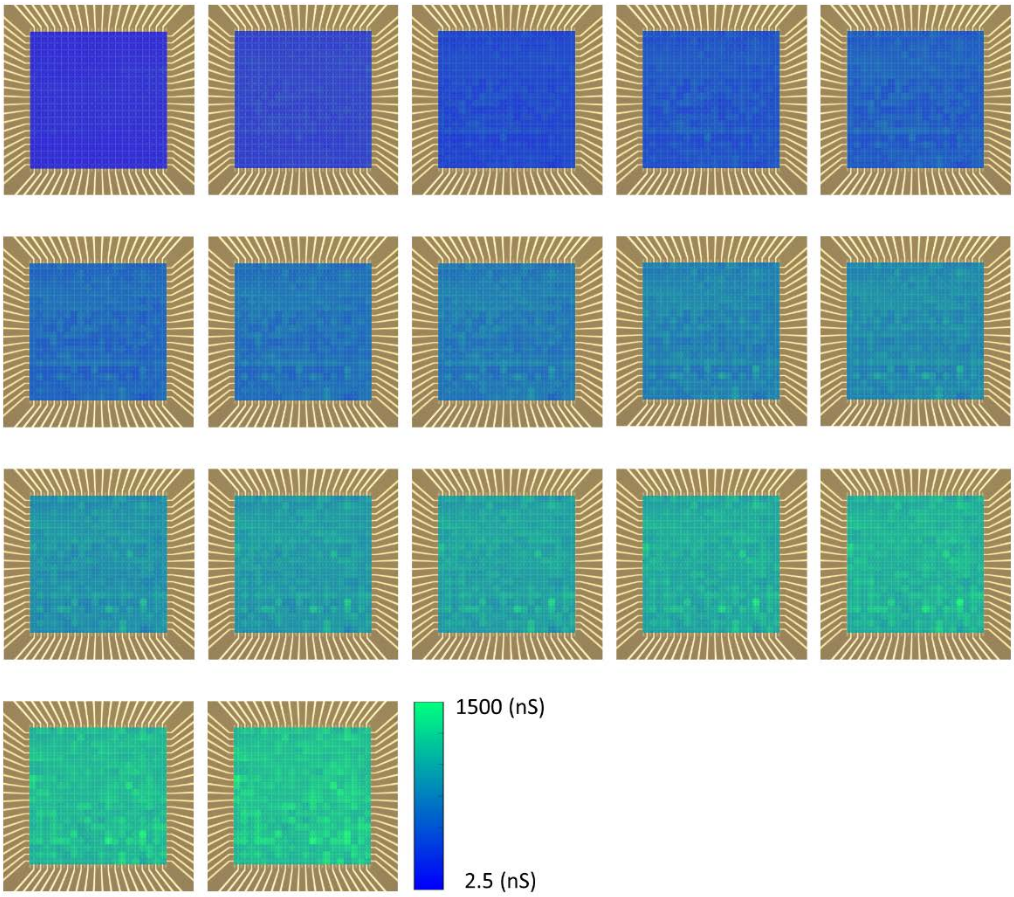

Supplementary Figure 4. Superior analog conductance change of gradual TiO_x memristor along with 16 SET pulses. The pulsed responses of every device in a 20×20 gradual TiO_x memristor cross-bar array are measured by using 16 SET pulses (4.5 V, 100 µs) and following self-decaying. The read pulse amplitude is 2 V and the width is 100 µs. Through the whole SET pulses, each memristor shows identical analog conductance change, which shows the superior uniformity of the gradual TiO_x memristor cross-bar array. The average on/off ratio is 22.

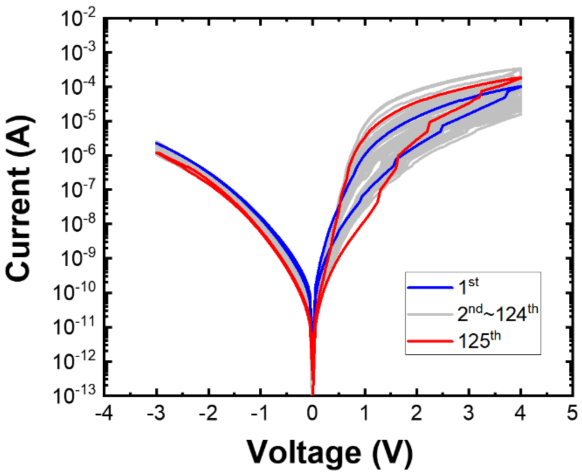

Supplementary Figure 5. Consecutive 125 I-V curves of the Pt/sputtered TiO_2/Ti device. Instead of the anodized TiO_x layer, RF-sputtered TiO_x layer by sputtering a TiO_2 target at room temperature is utilized as insulating material to study the effect of the anodizing process. The thickness of sputtered TiO_x layer and anodized layer are both about 30 nm. Unlike the anodized device, the sputtered device shows unstable resistive switching with high leakage current and large variation. The leakage current increases in the sputtered device and the self-rectifying property deteriorates. In addition to the leakage, the large variation and the poor uniformity are induced from the evenly distributed oxygen in the sputtered TiO_x. It is hard to modulate the effective insulator thickness, because more oxygen should be moved to reduce the effective insulator in sputtered TiO_x case. Therefore, the sputtered device shows a small on/off ratio at the first cycle. After applying 125 voltage sweeps, the sputtered device shows a high on/off ratio similar to the anodized device. The consecutive voltage stimulus might change the oxygen distribution, which has a high oxygen concentration at the top electrode side and a low oxygen concentration at the bottom electrode side, similar to the anodized device.

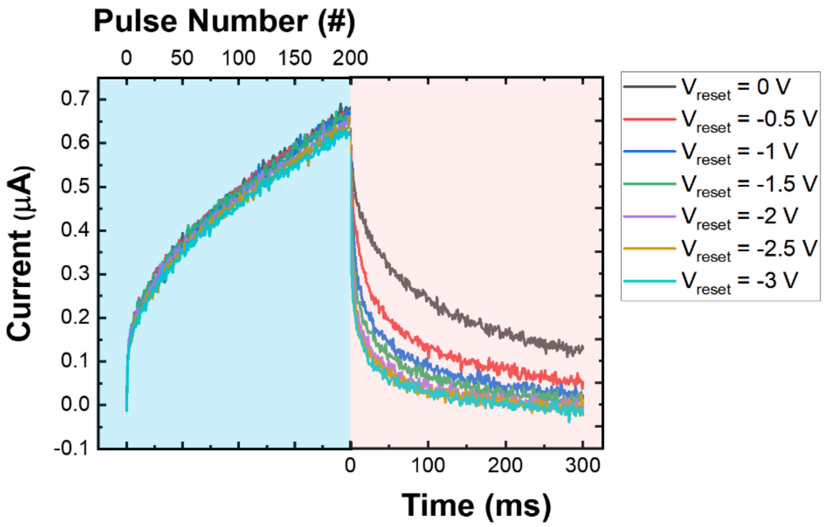

Supplementary Figure 6. Effect of RESET pulses in the self-decaying stage. A gradual TiO_x memristor is tested with 200 SET pulses (4 V, 20 μs) and is read by read pulse (1.5 V, 300 μs) as shown in the blue side of the figure. The RESET voltage is applied to show the effect of RESET bias when the device's conductance is decayed, as shown in the red side of the figure. The RESET pulse accelerates the decaying speed, and the larger RESET bias makes the decaying even faster than the small RESET bias. The RESET bias can be utilized in the neuro-memristive computing system where the memristors must be quickly initialized to process the next data.

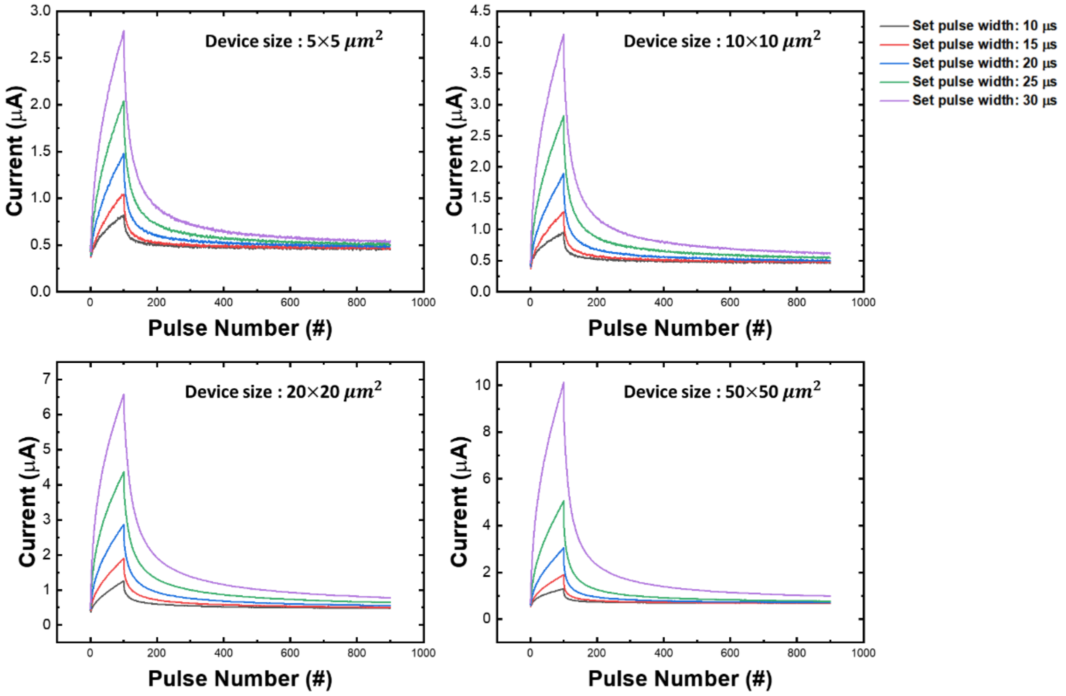

Supplementary Figure 7. The response of devices with different device sizes and set pulse widths. Four devices having different sizes from 5×5 μm^2 to 50×50 μm^2 are measured by the read pulses (1.8 V, 400 μs) during the consecutive 100 set pulses (4 V) and following decaying. The pulse widths are varied from 10~30 μs and the corresponding pulse intervals are 90~70 μs. The responses from each device are identical except the current scaling effect due to device size difference.

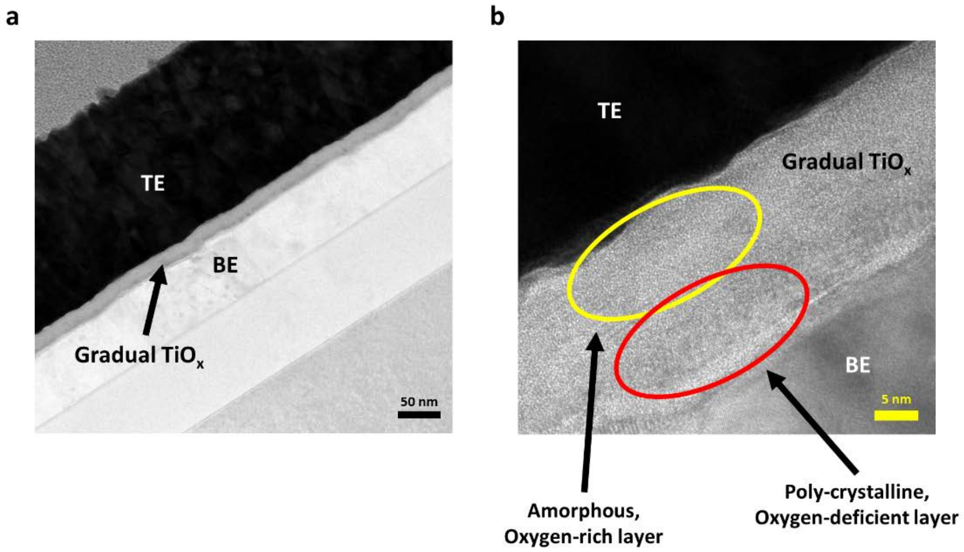

Supplementary Figure 8. TEM image of the gradual TiO$_x$ memristor. (a) The cross-section TEM image of the gradual TiO$_x$ memristor. The gradual TiO$_x$ layer is sandwiched between the BE and TE. The difference in the gradual TiO$_x$ layer between the TE side and BE side is easily observed. **(b)** The clearer image for gradual TiO$_x$ layer.

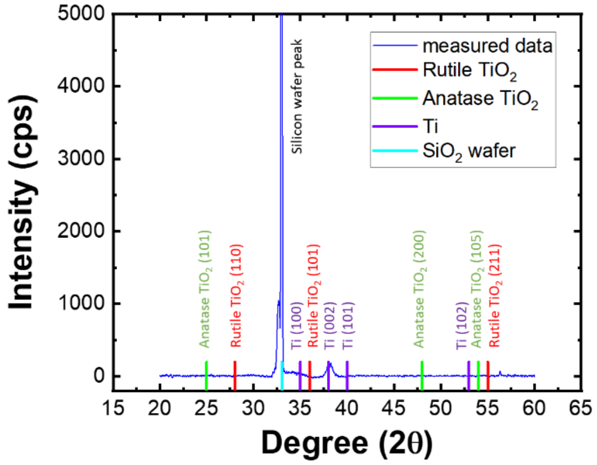

Supplementary Figure 9. The results of the X-ray diffraction analysis (XRD) for the anodized TiO_x layer. The X-ray is illuminated on the surface of the layer; thus, the information about the surface structure is revealed. The results show that the anodized TiO_x has a fully amorphous structure near the surface region, by showing that no pick related to the crystal TiO_2 (e.g. rutile or anatase) is observed in the measured data.

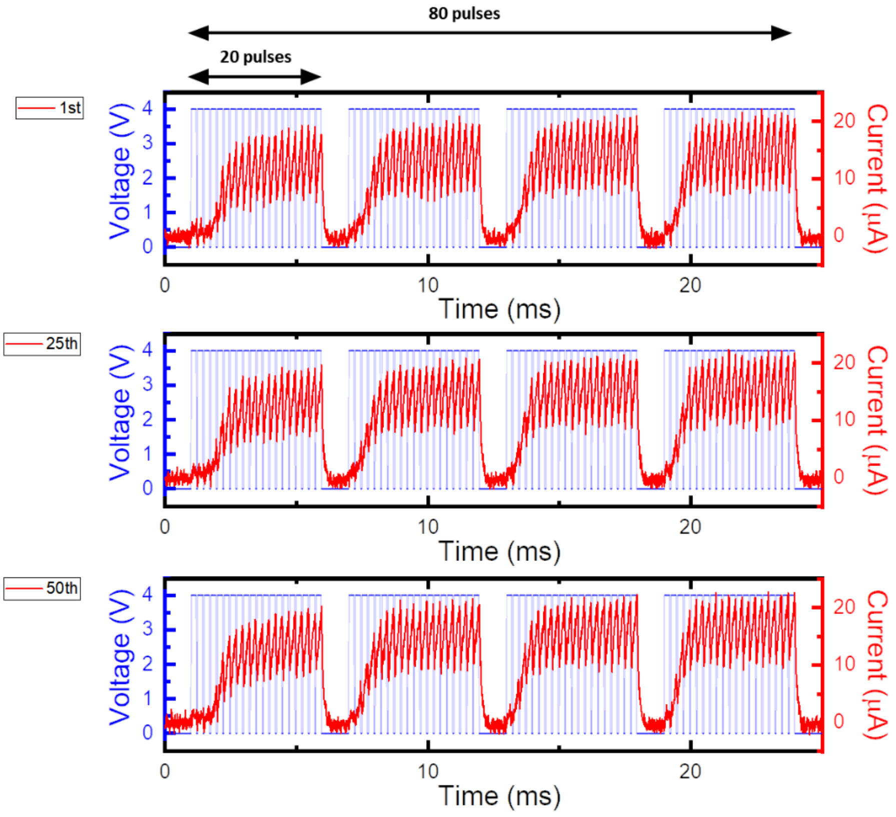

Supplementary Figure 10. Responses of the artificial neuron with several cycles of pulses. The artificial neuron is tested with an aggressive pulse train, where a single pulse train consists of 4 sub-pulse train, and a sub-pulse train has 20 pulses (4 V, 100 μs, and 50 μs interval). For one cycle, four sub-pulse trains are applied to the neuron with a 1 ms interval. The artificial neuron receives 50 cycles of the pulse train, which means that the neuron receives 4,000 pulses. During the test, the responses of the artificial neuron are not changed. The interval between each cycle is one-second, and the neuron goes back to the original state (memristor is fully reset) during the interval.

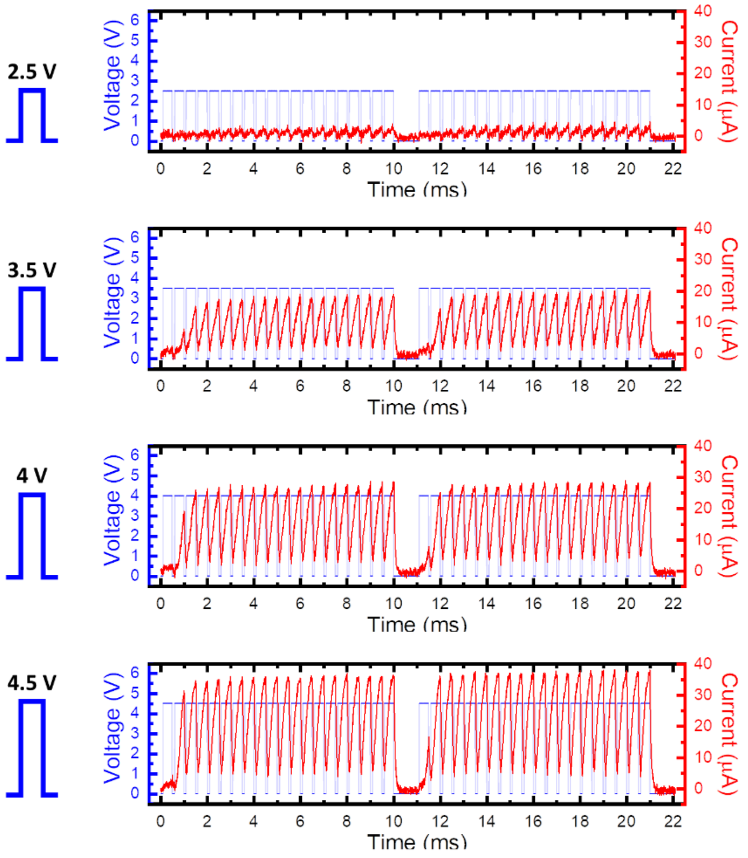

Supplementary Figure 11. The effect of the presynaptic spike amplitude to the artificial neuron response. The responses of the artificial neuron with different presynaptic spike amplitudes (2.5, 3.5, 4, and 4.5 V) are measured. When the spike amplitude is low (2.5 V), the artificial neuron does not fire, because the charges integrated in the capacitor are not enough to make high voltage that can turn the memristor on. However, when the spike amplitude is larger than 3.5 V, the neuron fires after integrating one or two presynaptic spikes. This result shows the artificial neuron can be modulated by the spike amplitude. The 400 μs spike width with 100 μs interval is used for all cases.

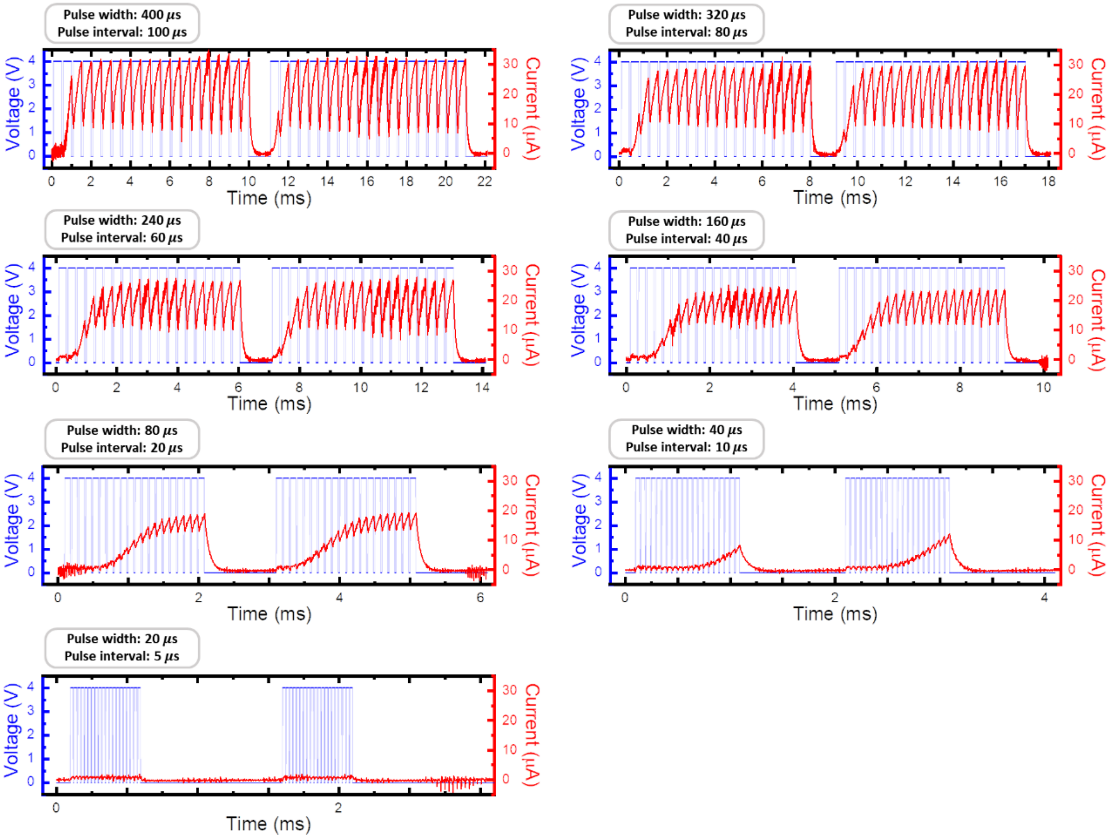

Supplementary Figure 12. The effects of the pulse (spike) width to the operation of the artificial neuron. The presynaptic spike width and interval are decreased in the same ratio, from 400 μs to 20 μs for spike width and from 100 μs to 5 μs for spike interval. From the results, it is demonstrated that the neuron operates with short pulses as short as 40 μs. During the test, the ratio between spike width and spike interval keeps constant.

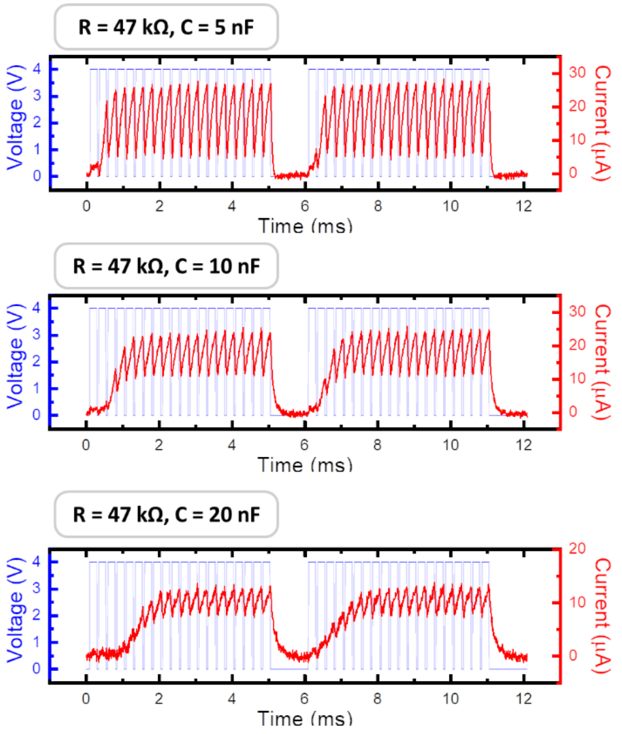

Supplementary Figure 13. Modulation of the threshold characteristic of the artificial neuron by changing the capacitance. Artificial neurons with various capacitance (5, 10, 20 nF) are developed to test the threshold modulation of the neuron. When the capacitance is small, the capacitor's potential, which is an analogy to the membrane potential of the biological neuron, is easily elevated up to the threshold voltage. Therefore, when a 5 nF capacitor is used in the artificial neuron, then the neuron fires after integrating a single presynaptic spike. However, if the capacitance increases into 10 nF, it requires two pulses to fire the post-synaptic spikes. Finally, when the capacitance is 20 nF, the neuron requires five spikes to fire. These results demonstrate that the developed artificial neuron can be easily tuned by modifying the capacitor.

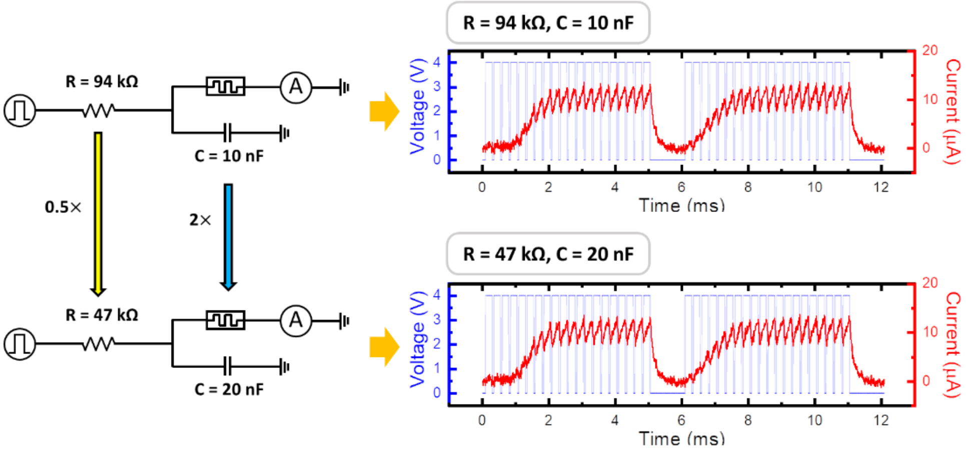

Supplementary Figure 14. The effect of the resistor and capacitor to the artificial neuron's characteristic. When two neurons have different resistance and capacitance but the RC value remains same, the neuron shows same responses. This is because the threshold voltage is determined by the charges in the capacitor, and the speed of charging the capacitor is represented as R×C. In the experiments, an artificial neuron is composed of 94 kΩ resistance and 10 nF capacitance (a neuron that easily fires but has a weak synapse), and the other is composed of 47 kΩ resistance and 20 nF capacitance (a neuron that hardly fires but has a strong synapse). The neurons show similar response to the presynaptic spike trains. The 4 V, 200 μs spike is used with 50 μs interval for the experiment.

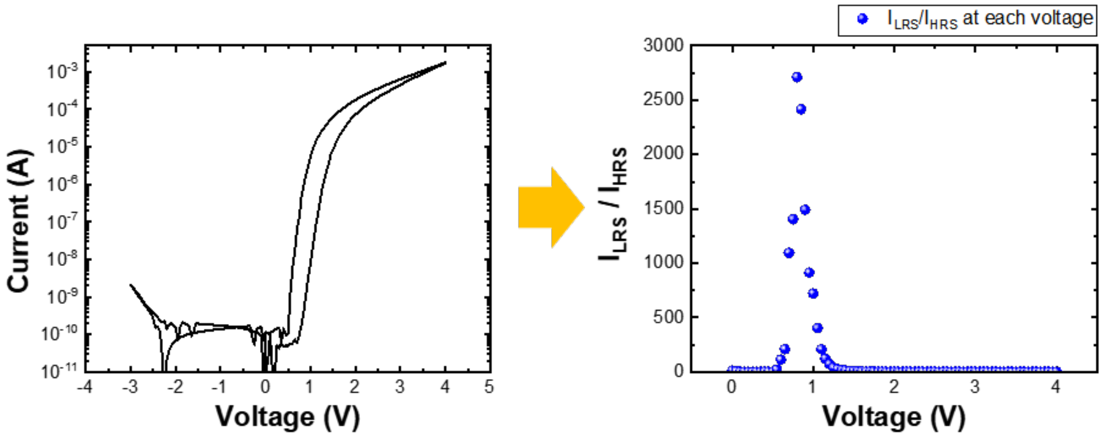

Supplementary Figure 15. A device I-V hysteresis curve (left), and an extracted on/off ratio of the device for each voltage point from the I-V curve (right). To investigate the maximum achievable on/off ratio of the device, the on/off ratio is extracted from the I-V curve of the device. The on/off ratio has the highest value (>2,700) when about 1 V is applied to the device.

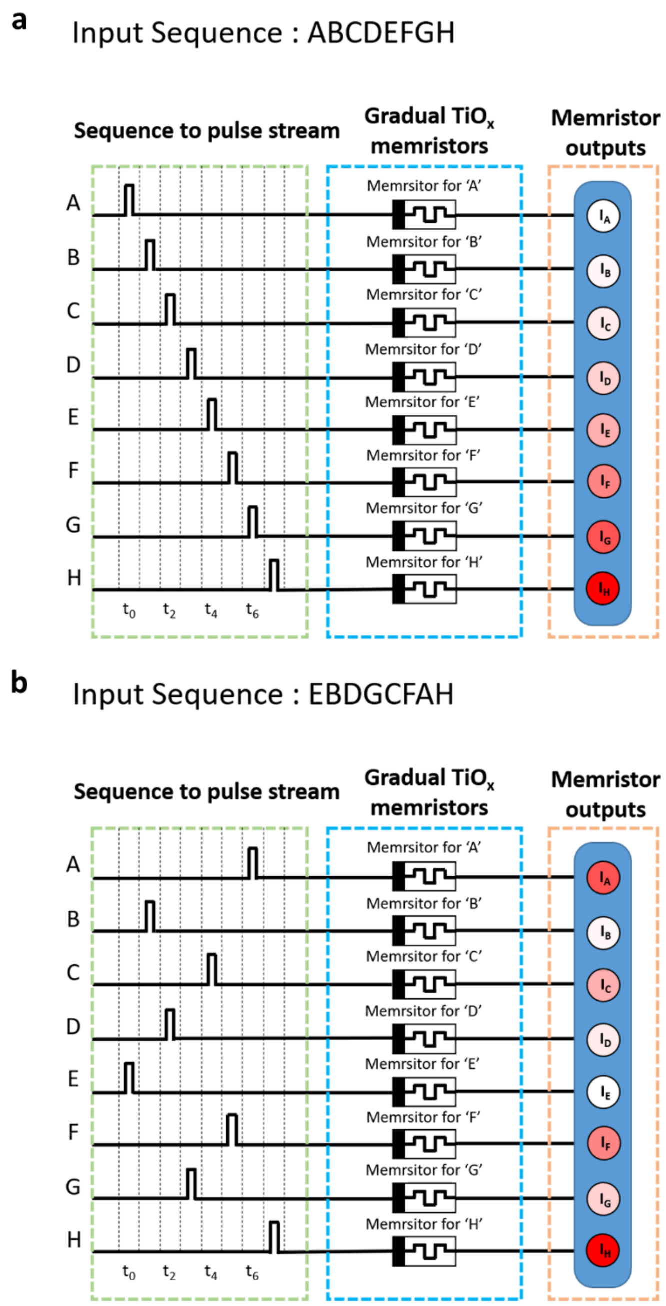

Supplementary Figure 16. A schematic of the memristors processing two different sequence input. (a) Input 'ABCDEFGH' feed into the memristors. Each alphabet in the input data is assigned to the memristor in a cross-bar array. When the corresponding alphabet is received, a SET pulse is applied to the assigned memristor. The states of the memristors are read for every time step. The memristor for an earlier entering element has lower conductance, because of the self-decaying property. **(b)** Input 'EBDGCFAH', which has different order but identical elements, feed into the memristors. Due to the self-decaying property of the gradual TiO_x memristor, the memristors can distinguish two different alphabet sequences from the output.

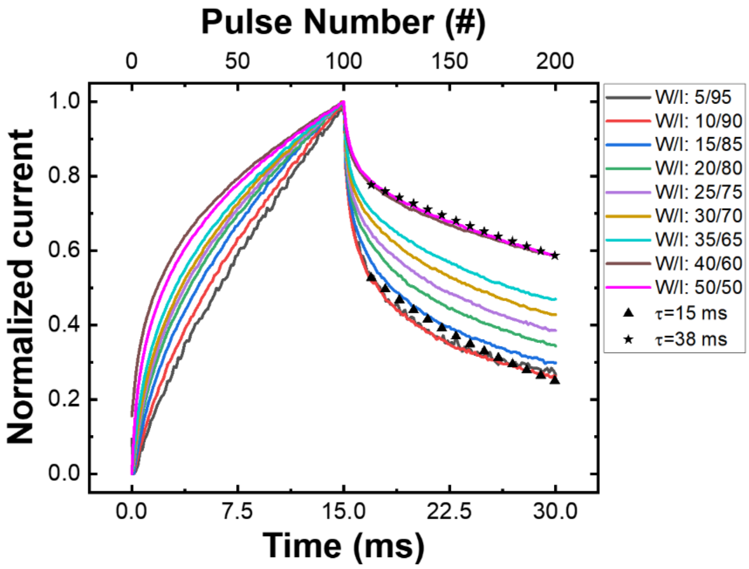

Supplementary Figure 17. The normalized responses of the gradual TiO_x memristor during the potentiation and decaying with various W/I ratios. To investigate the effect of the W/I ratio to the decaying time constant of the device, the gradual TiO_x memristor is potentiated with various W/I ratios from 5/95 to 50/50, and the decaying time constant is analyzed. The decaying time constant increases as the W/I ratio increases. The time constant is saturated to about 15 ms when the W/I ratio is smaller than 10/90. Similarly, the time constant is saturated to about 40 ms when the W/I ratio is larger than 40/60. The minimum and the maximum time constants are ~ 15 ms and ~ 40 ms, respectively. The W/I ratio that makes the time constant saturated can be different according to the pulse amplitude and the unit pulse width.

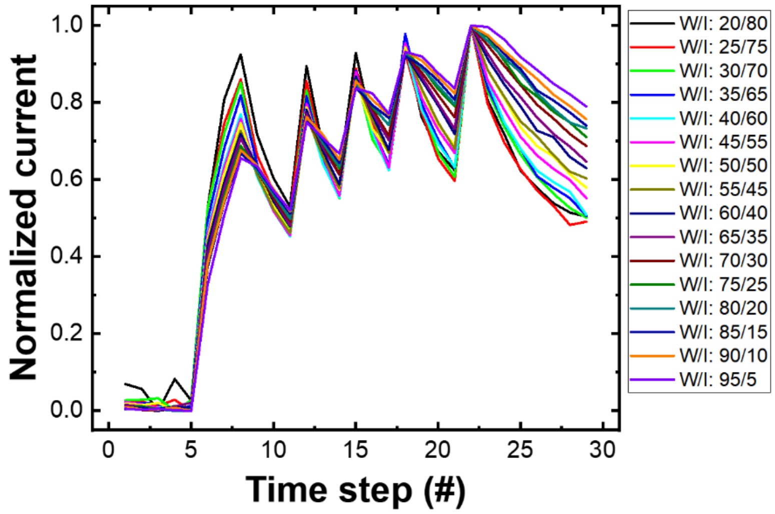

Supplementary Figure 18. The normalized memristor output current according to the binary pulse train with various W/I ratios. The unit pulse length is 100 μs, and the W/I ratio increases from 20/80 to 95/5 with a 5 μs interval. When the W/I ratio is low, the potentiation and decaying are abrupt. On the contrary, the potentiation and decaying are gradual in the high W/I ratio cases. The results show that memristors with different W/I ratios process the same sequential input differently, because of the different potentiation ratios and the decaying speed.

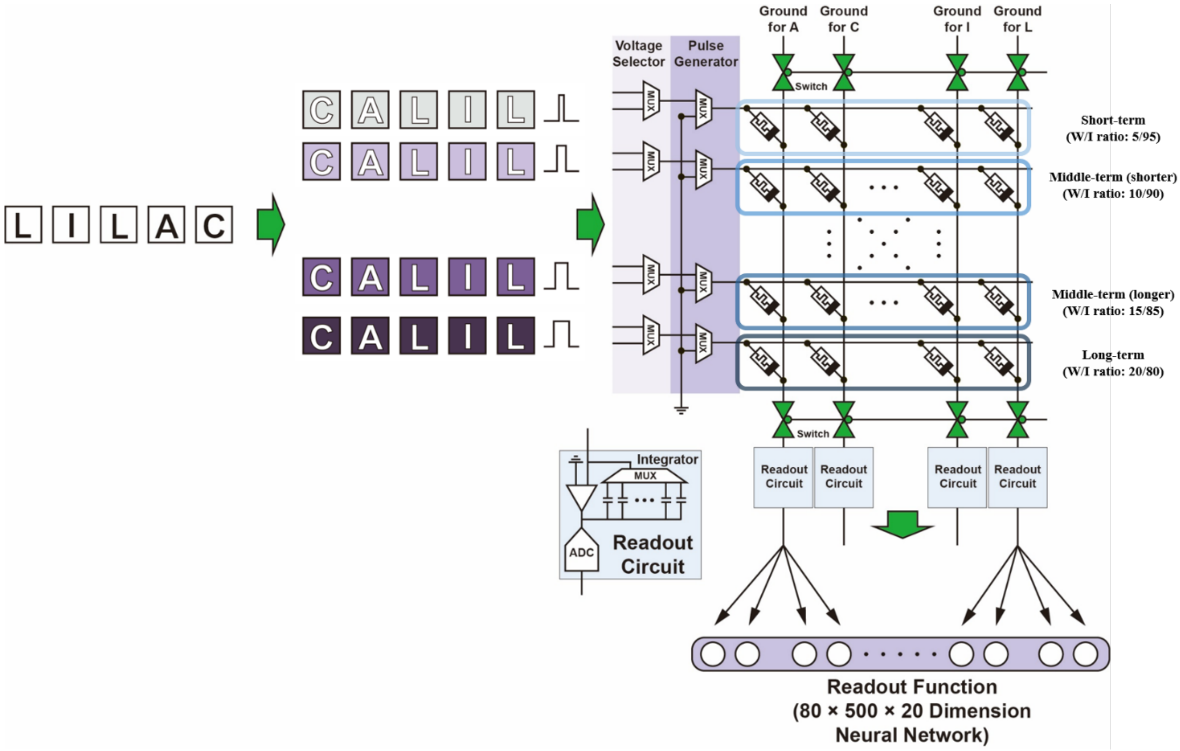

Supplementary Figure 19. A circuit schematic for the neuro-memristive computing system composed of the highly reliable dynamic memristor array and the peripheral circuit. Each sequence element (character) is assigned to a different column of the cross-bar array. Memristors in a same row of the cross-bar array shares a same W/I ratio (duty cycle), and memristors in different rows have different W/I ratios. A sequence enters each row of the memristor cross-bar array in form of a pulse train. The sequence data is transformed to the pulse train of desired duty cycles for each row by the pulse generator. The high voltage (amplitude) of the pulse is determined to the set voltage and the read voltage by the voltage selector. The selected column that is assigned to a certain character is grounded and the others are floated by the switches connected to the columns of the cross-bar array. The readout circuit is composed of an integrator and an analog-digital converter (ADC) and is connected to every column after the switch. The outputs from each memristor device are mapped to each node of the readout function, and the readout function trains its weights with backpropagation method based on the error between the readout function output and the target data.

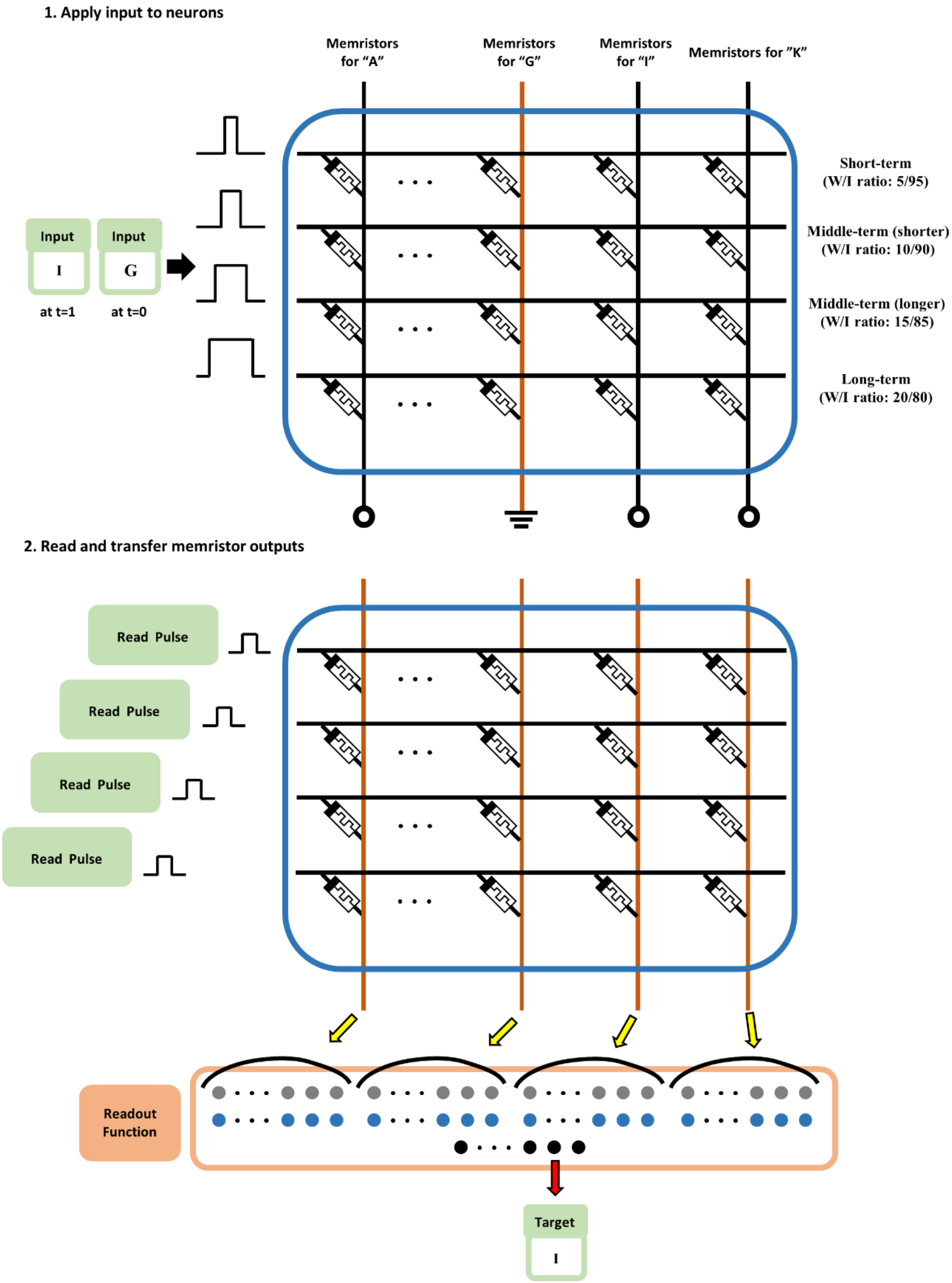

Supplementary Figure 20. An illustration of the operation of the developed neuro-memristive computing system. In the given example, input character "G" enters the cross-bar array and the column for the "G" in the array is grounded to potentiate the column "G". The pulse duty cycle for each row in the array is different to make the memristor to process the input differently. When the potentiation of the column finishes, the states of the memristors are

read through the read pulses through the row lines while the column lines are grounded. Then, the conductance of the memristors in the array are transferred to the input of the readout function. Based on the error between the readout function's output and the target "I", which is the next input of the input "G", the readout function trains its weights. The same processes repeatedly progressed until the end-cursor enters the system.

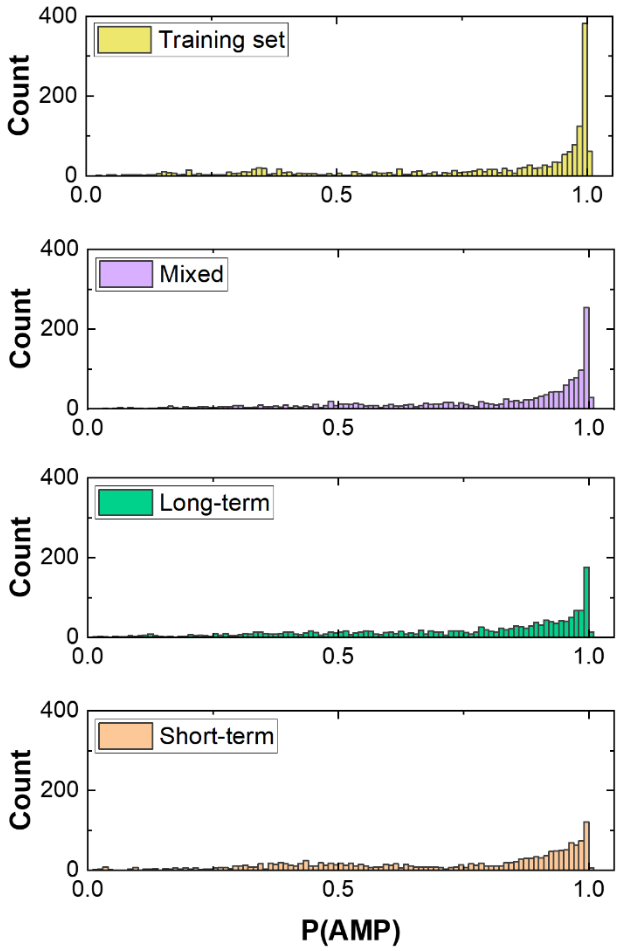

Supplementary Figure 21. The results of AMP prediction tool (CAMP). The results of AMP prediction tool about training set, generation sets from the mixed case (various W/I ratios), short-term (low W/I ratio), and long-term (high W/I ratio) cases respectively. P(AMP) is a probability of a sequence being AMP. When the P(AMP) of a sequence is bigger than 0.5, that sequence is considered as an AMP. The mixed case generates much more sequences with P(AMP)>0.5 compared to the short-term or long-term cases.

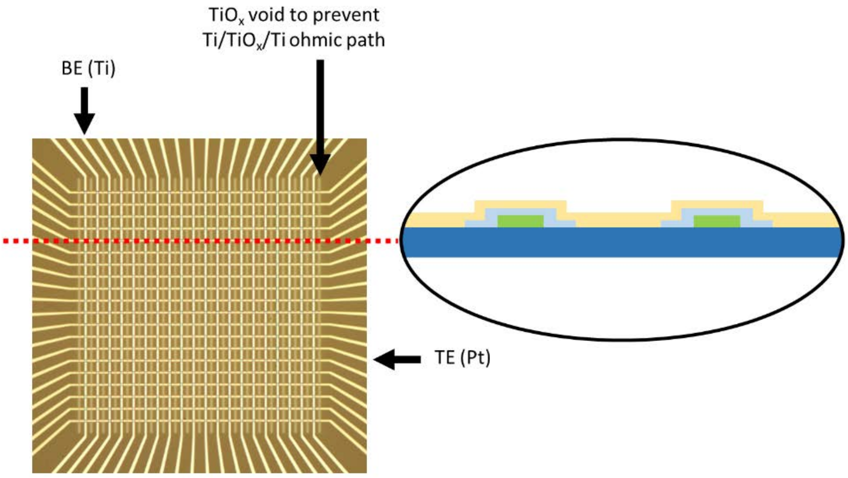

Supplementary Figure 22. The 20×20 gradual TiO$_x$ memristor cross-bar array. A top-view optical microscopy image of the cross-bar array. The inset shows the schematic of the cross-section of the cross-bar array. The gradual TiO$_x$ is patterned to disconnect Ti/TiO$_x$/Ti ohmic path, which causes cell-to-cell interference.

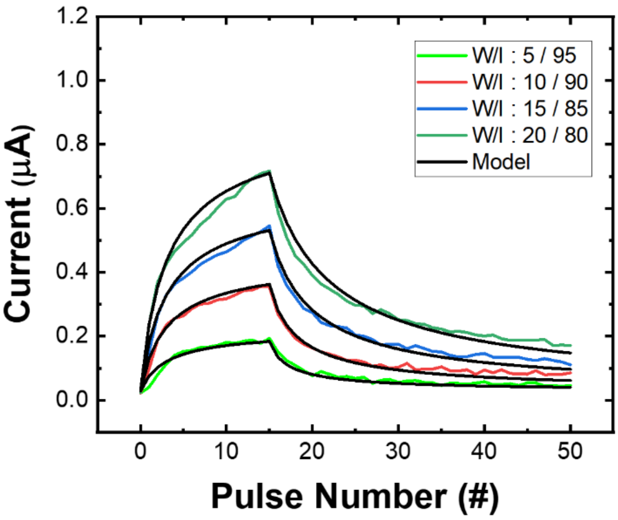

Supplementary Figure 23. The memristor modeling results. Pulsed responses of a gradual TiO$_x$ memristor with various W/I ratios are modeled to simulate the neuro-memristive computing system. The above 4 W/I ratios and the corresponding models are used for the simulation. The SET voltage is 4 V and the pulse width is controlled by modifying the duty cycle from 5 to 25 among the total of 100 μs.

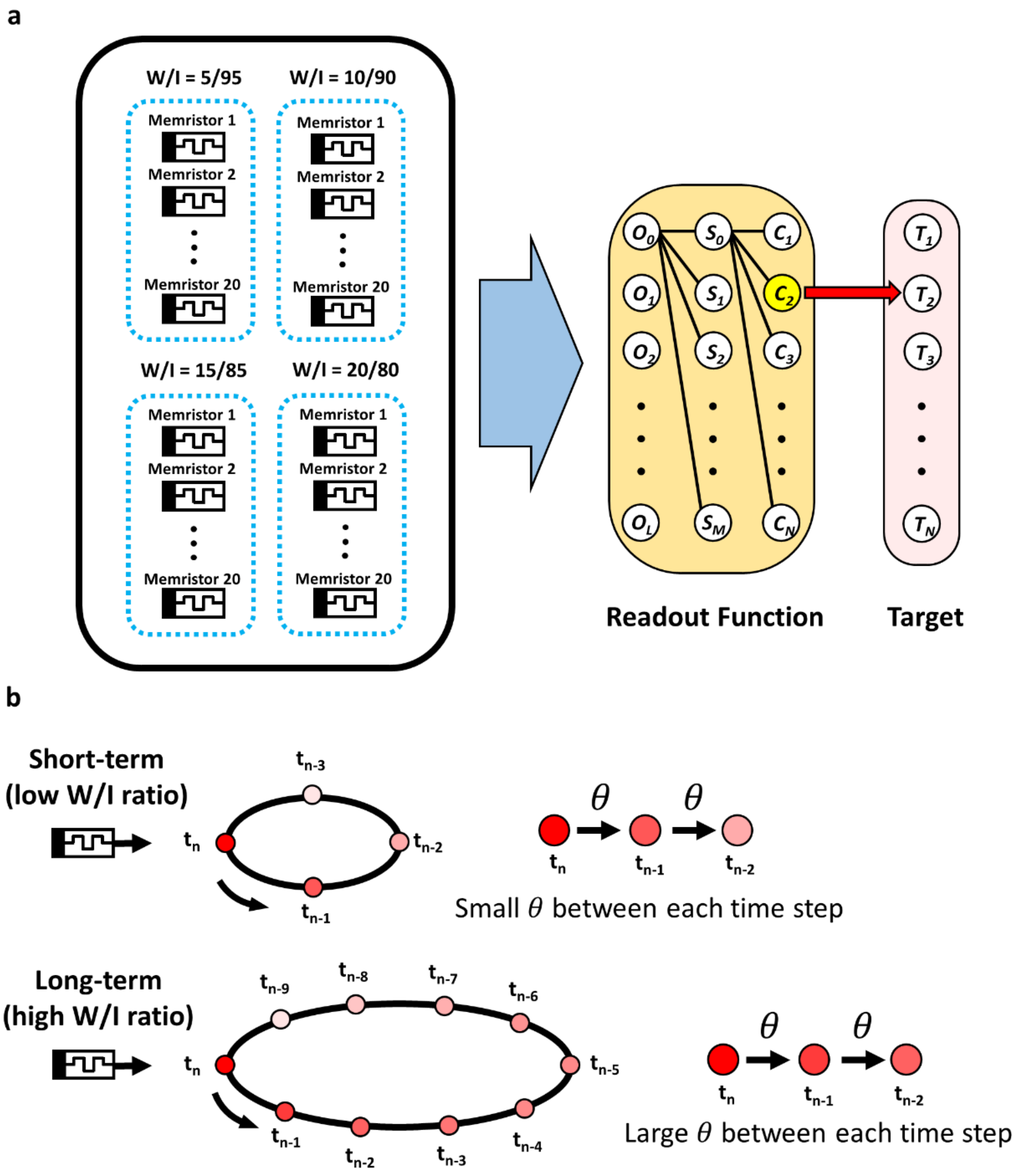

Supplementary Figure 24. Schematic of the neuro-memristive computing system with mixed W/I ratios. (a) The schematic of a neuro-memristive computing system with four groups of memristors which have different pulse W/I ratios. The memristors with a high W/I ratio process the input data in a long time window, while the memristors having a low W/I ratio process the input only in the short time window. Each element of the input (such as alphabet) is assigned to the one memristor for each W/I ratio group, thus total four memristors are assigned for each element in this case. The memristor outputs are feed into the readout function, and the readout function trains its trainable parameters through backpropagation. **(b)** The schematic representing the processing abilities in terms of weights between each time step according to the W/I ratios. In the short-term case (low W/I ratio), the weight (θ) between each time step is small, while it is much larger in the long-term case (high W/I ratio). The time between each time step is 100 μs in both short-term and long-term cases.

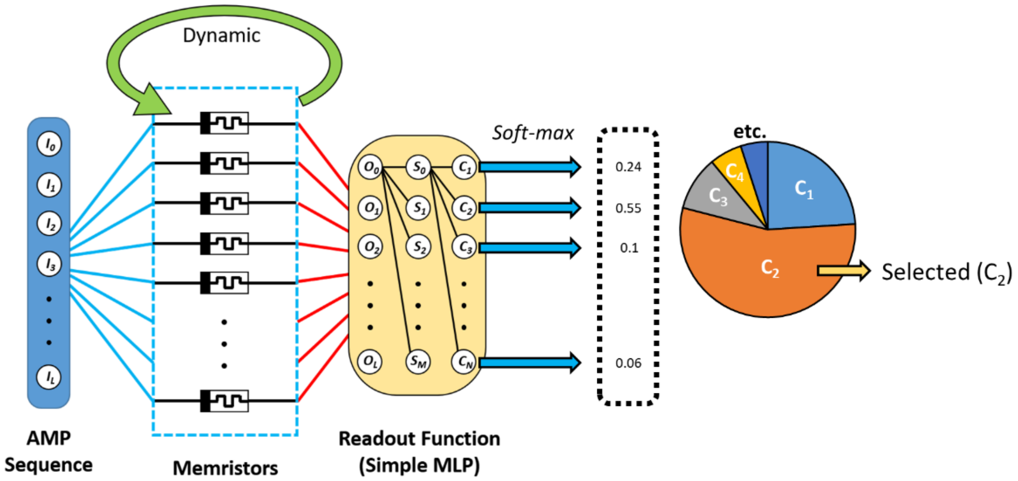

$$\text{Soft-max}: f(x_i) = \frac{e^{x_i}}{\sum_{k=1}^{N} e^{x_k}} \text{ for k = 1, ..., N}$$

Supplementary Figure 25. Schematic of the selection of the next amino-acid based on the soft-max output. The 3rd layer of the readout function makes output value using the soft-max function as an activation function. A soft-max is used to make the readout function output values as probabilities of each amino-acid being selected. The system selects the next amino-acid by making the random selection based on the probability from readout function output. This method gives variation to the generated sequences and prevents the neuro-memristive system from making the sequences already existing in the training set.

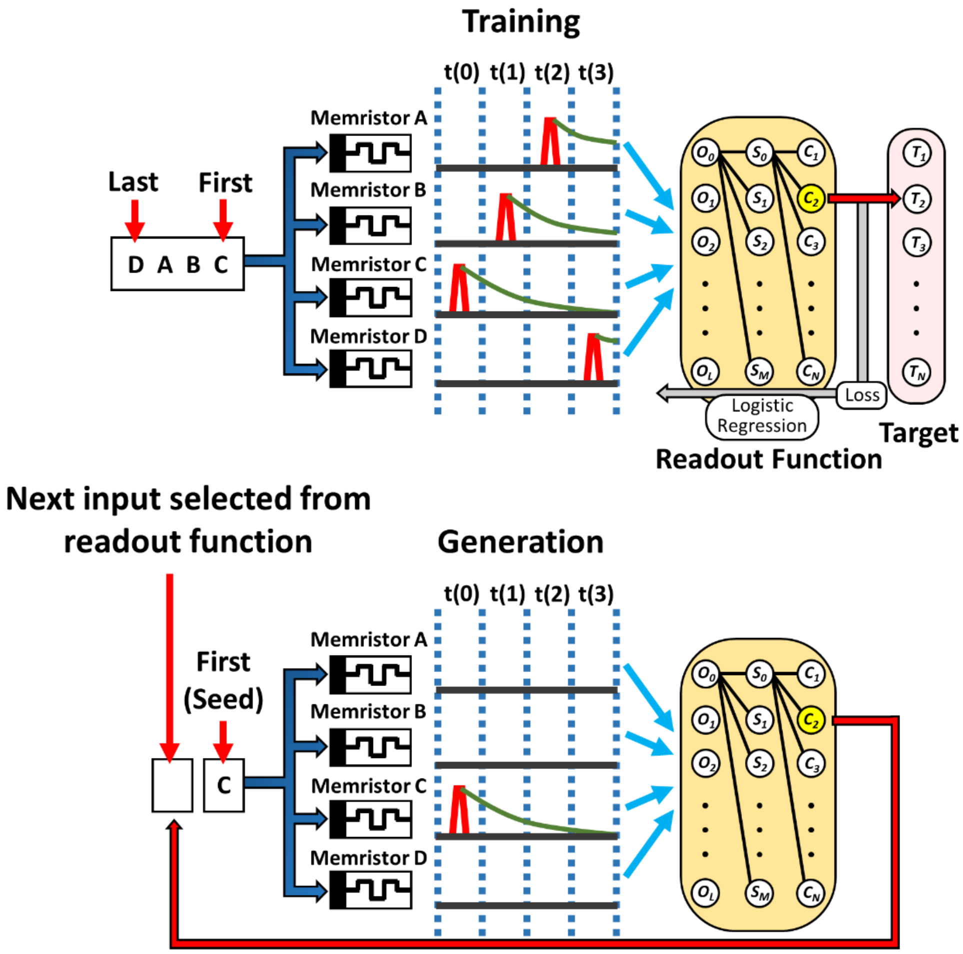

Supplementary Figure 26. Schematic of the neuro-memristive computing system operation at training phase and generation phase. At a training phase, the system learns the amino-acid grammar by predicting the next amino-acid. A conventional machine learning algorithm (logistic regression) is used for the training, based on the error (loss) between the target and selected amino-acid. At a generation phase, the system predicts the next amino-acid based on the trained information. The selected amino-acid becomes the next input of the system. The schematic does not consider the mixed case.

Device structure	Operation mechanism	Cycle-to-cycle variation	Device-to-device variation	Endurance	Spike peak current (μA)	Power at spike peak (μW)	Off resistance (R_{off}) (MΩ)
**Pt/gradual oxygen concentrationTiO$_x$/Ti**	**Ionic**	**1.39%**	**3.87%**	**> 5×10^6**	**10~35**	**35**	**100**
Pt/SiO$_x$N$_y$:Ag/Pt[1,2]	Ionic (diffusion of Ag)	N/A	N/A	N/A	20	10	200
Ag/SiO$_2$/Au[3]	Ionic (diffusion of Ag)	N/A	N/A	N/A	23	0.5	10,000
Pt/TiN/NbO$_2$/TiN/W[4]	Mott	Good, but not quantitively analyzed	N/A	> 10^6	1,500	133	N/A
Pt/Ti/NbO$_x$/Pt/Ti[5]	Mott	Good, but not quantitively analyzed	N/A	> 10^9	800	392	0.1
Pt/VO$_2$/Pt[6]	Mott	Good, but not quantitively analyzed	7%	> 26.6×10^6	> 60	11	0.01
TE/VO$_2$/BE[7]	Mott	N/A	N/A	10^9	200	11.9	0.1

Supplementary Table 1. Comparisons with various memristor-based artificial neurons. The cycle-to-cycle and device-to-device uniformities of the gradual TiO_X memristor are compared to the other existing neuron memristors (diffusive memristors and Mott memristors). The gradual TiO_X memristor shows superior uniformities in both cycle-to-cycle and device-to-device compared to the other neuron memristors. In addition, the gradual TiO_X memristor can be integrated in the cross-bar array form thanks to its superior reliability, while the others hardly show cross-bar array integration.

Device structure	Array size	On/off ratio	Cycle-to-cycle variation	Device-to-device variation	Pulse amplitude and width	Endurance	Rectification ratio
**Pt/gradual oxygen concentrationTiO$_x$/Ti**	**20 × 20 Crossbar array**	**> 2,000**	**1.39%**	**3.87%**	**Write : 4.5 V, 10 μs Read : 1.5 V, 100 μs**	**> 5×10^6**	**10^4**
Au/Pd/Wo$_x$/W[8]	32 × 32 Crossbar array	N/A	N/A	N/A	Write : 1.5 V, 1 ms Read : 0.5 V, 500 μs	N/A	N/A
Au/Pd/Wo$_x$/W[9]	32 × 32 Crossbar array	N/A	N/A	N/A	Write : 3 V, 10 μs Read : 0.6 V, 200 μs	N/A	N/A
Pt/SiO$_x$N$_y$:Ag/Pt[10]	Stand-alone	> 10^4	N/A	N/A	Write : 1.25 V, 100 μs Read : 0.1 V, 300 μs	> 10^6	N/A
Pt/TaO$_y$/TiO$_x$/Ti[11]	Stand-alone	N/A	Good, but not quantitively analyzed	N/A	Write : -2 ~ 2 V, 120 μs	N/A	10^3
Au/Cr/SnS flake/Au/Cr[12]	1 × 10 array	< 2	N/A	N/A	Write : 4.5 V, 20 ms Read : 1 V, 25 ms	N/A	N/A

Supplementary Table 2. Comparisons with other various memristors for neuro-memristive computing. To demonstrate the effectiveness of the gradual TiO_X memristor for neuro-memristive computing system, several characteristics of the device are compared to the various memristors used for neuro-memristive computing system. The gradual TiO_X memristor satisfies the superior uniformity, high on/off ratio, fast speed, high endurance, and high rectifying ratio for sneak path problems simultaneously.

Supplementary References:

1. Wang, Z. *et al.* Fully memristive neural networks for pattern classification with unsupervised learning. *Nat. Electron.* **1**, 137–145 (2018).

2. Wang, Z. *et al.* Memristors with diffusive dynamics as synaptic emulators for neuromorphic computing. *Nat. Mater.* **16**, 101–108 (2017).

3. Zhang, X. *et al.* An Artificial Neuron Based on a Threshold Switching Memristor. *IEEE Electron Device Lett.* **39**, 308–311 (2018).

4. Kumar, S., Williams, R. S. & Wang, Z. Third-order nanocircuit elements for neuromorphic engineering. *Nature* **585**, 518–523 (2020).

5. Duan, Q. *et al.* Spiking neurons with spatiotemporal dynamics and gain modulation for monolithically integrated memristive neural networks. *Nat. Commun.* **11**, 1–13 (2020).

6. Yi, W. *et al.* Biological plausibility and stochasticity in scalable VO_2 active memristor neurons. *Nat. Commun.* **9**, (2018).

7. Jerry, M., Parihar, A., Grisafe, B., Raychowdhury, A. & Datta, S. Ultra-low power probabilistic IMT neurons for stochastic sampling machines. *IEEE Symp. VLSI Circuits, Dig. Tech. Pap.* **8**, T186–T187 (2017).

8. Du, C. *et al.* Reservoir computing using dynamic memristors for temporal information processing. *Nat. Commun.* **8**, 1–10 (2017).

9. Moon, J. *et al.* Temporal data classification and forecasting using a memristor-based reservoir computing system. *Nat. Electron.* **2**, 480–487 (2019).

10. Midya, R. *et al.* Reservoir Computing Using Diffusive Memristors. *Adv. Intell. Syst.* **1**, 1900084 (2019).

11. Zhong, Y. *et al.* Dynamic memristor-based reservoir computing for high-efficiency temporal signal processing. *Nat. Commun.* **12**, 1–9 (2021).

12. Sun, L. *et al.* In-sensor reservoir computing for language learning via two-dimensional memristors. *Sci. Adv.* **7**, (2021).
